# Undescended Testes and Laparoscopy: Experience from the Developing World

**DOI:** 10.1155/2018/1620470

**Published:** 2018-10-24

**Authors:** Shabir Ahmad Dar, Rajandeep Singh Bali, Yawar Zahoor, Arshad Rashid Kema, Rajni Bhardwaj

**Affiliations:** ^1^MS General Surgery, Resident Surgeon, SKIMS, Jammu and Kashmir, India; ^2^MS General Surgery, Consultant Surgeon, GMC-Jammu, Jammu and Kashmir, India; ^3^MS General Surgery, Resident Surgeon, MAMC, Delhi, India; ^4^MS General Surgery, Consultant Surgeon, DHSK, Jammu and Kashmir, India

## Abstract

**Background:**

Cryptorchidism or undescended testes is the most common disorder of the male endocrine glands in children. With the advancements in laparoscopic techniques and instruments, laparoscopic orchidopexy has become the standard procedure in the management of nonpalpable undescended testes.

**Aim:**

To evaluate and determine the therapeutic role, sensitivity, and specificity of laparoscopy in localizing nonpalpable testes and the mean operative time, the conversion rate (and reasons thereof), postoperative wound infection, postoperative stay, and time taken for return to daily activities following laparoscopic orchidopexy or orchidectomy.

**Materials and Methods:**

This was a prospective study carried out in the Postgraduate Department of Surgery, Government Medical College, Srinagar, J&K, India, from May 2008 to August 2011. All patients who presented to the outpatient department with complaints of absent testes were examined, and the ones with nonpalpable testes were included in the study.

**Results:**

The mean operative time for bilateral and unilateral nonpalpable testis was 102.76 and 53.67 minutes, respectively. Minor postoperative wound infections were noted in 4 of our patients. Mean duration of hospital stay was 14.23 hrs for unilateral cases and 16.27 hrs for bilateral cases. Patients who underwent laparoscopic orchidopexy resumed their normal activities within 4 ± 1 days.

**Conclusion:**

Laparoscopy clearly demonstrates the anatomy and provides visual information upon which a definitive decision can be made for further management of the undescended nonpalpable testis.

## 1. Introduction

Cryptorchidism or undescended testes is the most common disorder of the male endocrine glands in children. Physical examination of the testis can be difficult, and further evaluation should be considered if a normal testis cannot be definitely identified. Reasons for treatment of cryptorchidism are increased infertility, testicular malignancy, testicular torsion, trauma, and psychological stigma of an empty scrotum [1].

About 1% incidence of cryptorchidism is reported by 1 year of age, and about 20% of children with cryptorchidism may have one or both nonpalpable testes. The condition of the nonpalpable testis may fall into one of the following categories: agenesis, vanishing testis, intra-abdominal testes, or inguinal testes [2]. Although there are various imaging studies, i.e., ultrasonography, CT, and MRI, have been used to locate nonpalpable testes in boys, none has been able to provide comparable accuracy to laparoscopy [3]. In experienced hands, laparoscopy is capable of providing 100% accuracy in the diagnosis of the intra-abdominal testis with minimal morbidity. With the advancements in laparoscopic techniques and instruments, laparoscopic orchidopexy has become a standard procedure in the management of nonpalpable undescended testes.

Approximately two-thirds of neonates born with an undescended testis will undergo spontaneous testicular descent, typically by 4 to 6 months postnatally, mediated by postnatal testosterone surge [4]. Approximately 20% of all cryptochoid testes are nonpalpable. Of all childhood testes that are nonpalpable, 50 to 60 percent are intra-abdominal but can also be found in the inguinal canal (canalicular) or just inside the internal ring (peeping) [5].

Majority of the intra-abdominal testes are located less than 2 cm from the internal ring. The intra-abdominal testis may be associated with a closed or an open deep inguinal ring. In the closed ring variant, the processus vaginalis does not develop, the gubernaculum is absent, and the internal ring is closed. In the open ring variant, a patent processus vaginalis exists in the internal inguinal ring and the gubernaculum is present. Vanishing testis is one in which testicular vessels and vas deferens are found on surgical exploration but a testis is absent. Supporting evidence for testicular infarction includes the common finding of hemosiderin, and calcium deposits in testicular remnants found on exploration [6]. One in 600 males has bilateral undescended testes, representing 10 to 25% of patients with cryptorchidism. The finding of bilateral nonpalpable testes however represents a special situation that may warrant further investigations to rule out intersex abnormalities which may have life-threatening implications in the neonatal period, especially associated with severe hypospadias.

## 2. Aim

This study entailed the following aims and objectives:

1. To determine the sensitivity and specificity of laparoscopy in localizing nonpalpable testes
2. To evaluate the therapeutic role of laparoscopy in nonpalpable testes
3. To determine the mean operative time, the conversion rate (and reasons thereof), postoperative wound infection, postoperative stay, and time taken for return to activities of daily life

## 3. Materials and Methods

**Figure 1:**
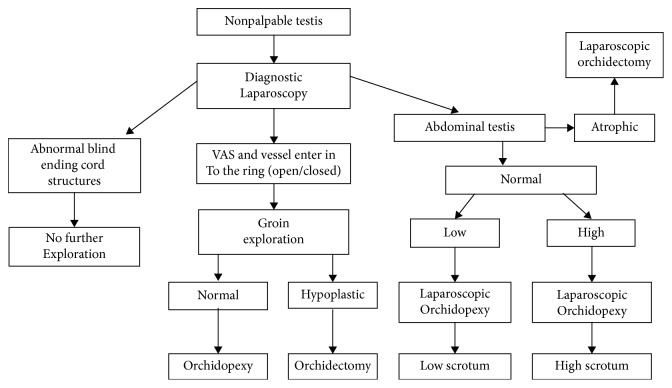
Algorithm for the management of nonpalpable undescended testes.

This was a prospective study carried out in the Postgraduate Department of Surgery, Government Medical College, Srinagar, from May 2008 to August 2011. All patients who presented to the outpatient department with complaints of absent testes were examined, and boys with nonpalpable testes were included in the study. These boys were examined as outpatients, at the time of admission and after general anesthesia to exclude palpable testes. Patients with nonpalpable testes were subjected to diagnostic and operative laparoscopy. The primary 5 mm camera port was introduced by open technique (inferior rim of the umbilicus on its internal surface), pneumoperitoneum was created (10 mmHg) with the patient in a 30° head-down Trendelenburg position, the area of the internal inguinal ring was inspected bilaterally, and subsequent trocars (all 5 mm) were placed under laparoscopic (30° telescope) visualization into the right and left lumbar region in line with the umbilical port. Subsequent surgery was planned according to the laparoscopic findings following the algorithm for the management of the nonpalpable testis (Figure 1).

A diagnostic laparoscopy was done for rest of the abdominal quadrants in all cases. The inguinal ring was first examined to evaluate its patency, and then the bilateral iliac areas and pelvis were inspected. If intra-abdominal blind ending cord structures were found, no further exploration was performed, and a diagnosis of intra-abdominal vanishing testes was made.

If an intra-abdominal testis was found, it was classified as high (in the iliac fossa or pelvic inlet or pelvis >2 cm from the internal ring) or low (adjacent to the internal ring/emergent testis <2 cm from the internal ring) depending on its position to the internal ring.

With gentle traction on the testis, the most distal gubernacular attachment was divided with electric hook cautery, and gubernaculum was used as a handle for mobilizing the testis. Once the testis had been adequately mobilized, confirmed by performing stretch maneuver where the testes are stretched to the contralateral deep inguinal ring, the dissection was further carried out medially all around the ipsilateral deep inguinal ring to complete the herniotomy. Peritoneal coverage between the vas deferens and urinary bladder was dissected. Usually, a neotunnel was created between the medial umbilical ligament and the inferior epigastric artery (Prentiss maneuver) to gain extra length for reaching the scrotum. The blunt tip of the laparoscopic dissector was passed over the pubic bone into the ipsilateral hemiscrotum, thus creating a pathway. A dartos pouch was prepared in the hemiscrotum. A suction cannula was passed along the passage which was created transabdominally by laparoscopic dissector, and grasping the tip of the suction cannula, a long curved clamp was passed into the abdominal cavity and stretched in all directions to create adequate passage for bringing the testes down. The gubernacular tissue was grasped and pulled, thus delivering the testes out into the scrotal dartos pouch, and the length was checked by deflating the abdomen. Releasing the pneumoperitoneum gave additional length. Any tension found on the pedicle was released by further mobilization of the peritoneal flaps. Testes were fixed in the scrotum using 5-0 chromic catgut suture. At the end of the procedure, the abdomen was examined for any bleeding, the pneumoperitoneum was released, and trocars were removed. Trocar sites were closed. Children were discharged home next day. All the patients were followed at 6 weeks and 6 months.

## 4. Results

We used diagnostic and operative laparoscopy in the management of 52 patients with 61 undescended testes. 26 patients had 28 palpable testes (45%), and 26 patients had 33 nonpalpable testes (55%). Boys with the palpable testis at any point were excluded from the study. The study sample size of our series was 26 patients with 33 nonpalpable testes. Ratio of palpable testis to nonpalpable testis was 1 : 1.17, respectively.

The youngest and the eldest patient in our study group were 1 and 18 years of age, respectively. Majority of patients, i.e., 12, were in the age group of 4–7 years. Of these 33 nonpalpable testes, 7 (21%) were on the right side, 12 (36%) on the left side, and 14 (42%) bilateral. On ultrasonography, nonpalpable testes were localized at various sites as intra-abdominally (3 cm proximal to deep inguinal ring), near deep inguinal ring, in inguinal canal, and in superficial inguinal pouch and could not be localized in 11 (33.33%), 9 (27.2%), 3 (9%), 3 (9%), and 7 (21.2%) cases, respectively.

Table 1 shows the relation of testis to deep inguinal ring as confirmed on diagnostic laparoscopy (*n*=33).

On diagnostic laparoscopy, the deep inguinal ring was found to be open or closed in 30 (90.9%) and 3 (9.1%) cases, respectively. The number of testes found normal on diagnostic laparoscopy was 18 (54.5%) followed by 11 hypoplastic (33.3%) and 3 atrophic (9.09%) testes. Blind ending vas deferens and vessels (vanishing testis) were found in 1 (3.03%) patient only.

Majority of high intra-abdominal testis were hypoplastic (58.8%), highlighting the deleterious effect of increased abdominal temperature on development of the testis.

Table 2 shows the morphology of the undescended nonpalpable testis in relation to its position as determined by diagnostic laparoscopy.

The operative procedures performed were laparoscopic orchidopexy, laparoscopic orchidectomy, open orchidopexy (conversion), open orchidectomy (conversion), and diagnostic laparoscopy in 24 (72.7%), 5 (15.1%), 2 (6.06%), and 1 (3.03%) cases, respectively. All of the patients were managed by single-stage procedure.

Laparoscopy was successful (diagnostic or therapeutic) in 30 (90.90%) cases, and the procedures included laparoscopic orchidopexy, laparoscopic orchidectomy, and diagnostic laparoscopy. Mean operative time for 14 bilateral undescended testes was 102.76 (with SD of 2.37 and SEM of 5.08) minutes while as for 19 unilateral cases, it was 53.67 (with SD of 5.38 and SEM of 4.02) minutes.

Postoperative complications included surgical emphysema [2], type 1 surgical site infection [1], and scrotal hematoma [1]. The average postoperative hospital stay was 14.23 hours (SD 2.37 hours) for unilateral undescended nonpalpable testes and 16.27 hours (SD 5.38 hours) for bilateral undescended nonpalpable testes. Most of our patients were discharged on next day because no immediate postoperative surgical complications were seen. Time taken to return to daily activities was more in patients who underwent orchidopexy (4 ± 1 days) as compared to patients who underwent orchidectomy (2 ± 1 days).

## 5. Discussion

The median age group of our study subjects was 5.2 years with patients from 1 to 18 years of age included in our study. Satar and Hvistendahl have reported similar median age group in their respective studies as 5.4 and 5.7 years [7, 8].

42% of our patients had bilateral undescended testis, and the incidence of bilateral undescended testis was relatively higher as compared to those reported by Ismail (13.5%), Zubair (33%), and Hassan (21%) [9–11]. This could be explained by the fact that our hospital is the main referral centre for laparoscopy and that our patients presented early as compared with other studies.

The USG (abdomen) could localize 78.78% of nonpalpable testes in our study. Ismail reported that USG was helpful in localization of the nonpalpable testis in 100% of his patients, as he used colour Doppler USG in his study [9].

We found the diagnostic yield of laparoscopy was 100% and the therapeutic yield was 96.9%, as we could localize and manage 32 nonpalpable testes while one case was diagnosed as vanishing testes on laparoscopy. El-Gohary has reported 100% accuracy in laparoscopic assessment of the testis [12].

The closed deep inguinal ring with vas deferens and vessels traversing it were found in 28% of our study subjects. The significance of this fact was that these patients had testis in the superficial inguinal pouch (ectopic testis). Godbole (33%) and Masao (35%) have also reported similar findings of the closed deep inguinal ring from their studies [13, 14].

Our study revealed normal testes in 54.5% of cases followed by hypoplastic testes in 33.3% of cases. Morphology of testis was correlated with localization of testis which revealed that features of hypoplasia were high in intra-abdominal testis. Similar findings were noted in the study of Humphery and Boekmann [5, 15]. One patient (3%) in our study had vanishing testis, confirmed upon diagnostic laparoscopy which saved the patient from unnecessary groin exploration, as has been suggested by Koyama et al. [2].

We performed orchidopexy in 26 out of 33 nonpalpable testes (78%) out of which laparoscopic orchidopexy was done in 24 (72.7%) testis, and conversion to open orchidopexy was necessitated in 2 (6.06%). The reasons for conversion were dense adhesions of testis with surrounding structures. Orchidectomy was done in 6 (18%), of which 3 testes were atrophic and 3 testes were hypoplastic (could not be mobilized adequately as determined by the stretch test). Masao and Khan et al. have reported laparoscopic orchidopexy in 33% and 35% cases [14, 16]. The high percentage of successful laparoscopic orchidopexy in our study can be explained by the fact that 96% of testis in our study were intra-abdominal or intracanalicular; moreover, the number of vanishing testis (3%) in our study group was much lower as reported by Humphrey (19%), Ismail (9%), and Hassan (38%) [5, 9, 11].

The mean operative time in our study was 53.67 minutes (SD of 2.37 minutes) for unilateral testis and 102.76 minutes (SD of 5.38 minutes) for bilateral nonpalpable testis. Mark and Davidson reported 15 minutes as average time for laparoscopy in unilateral nonpalpable testis [17]. As our study was the first of its kind at our institution and because of the learning curve of pediatric laparoscopy, operative time was relatively more in our study.

No major surgical complications were observed in our patients. Postoperative surgical emphysema was seen in 2 of our cases, which resolved spontaneously. One of our patients developed superficial surgical site infection, and one patient developed scrotal hematoma. The patients were on regular follow-up. No evidence of testicular atrophy was seen in those patients with follow-up till the study time. Various other authors have also reported similar findings as regards to complications following laparoscopic management of nonpalpable undescended testes [16, 18, 19]. This substantiates the fact that complication rates are markedly reduced in good centres with high expertise.

The average postoperative hospital stay was 14.23 hours (SD of 2.37 hours) for unilateral undescended nonpalpable testes and 16.27 hours (SD of 5.38 hours) for bilateral undescended nonpalpable testes. Most of our patients were discharged on the next day because no immediate postoperative surgical complications were seen. Time taken to return to daily activities was more in patients who underwent orchidopexy (4 ± 1 days) as compared to patients who underwent orchidectomy (2 ± 1 days). Koyama reported a hospital stay of less than one day, and Desai discharged his patients on the next day [2, 20].

In one (3%) of our cases, no testis was found as it was a vanishing testis. The morphology of testes as determined by laparoscopy was 96.9%. Laparoscopic procedures were performed in 90.9% of our cases. Open orchidectomy/orchidopexy was done in 9.1% of cases. Patients with vanishing testis (3%) had the advantage of avoiding unnecessary groin exploration. Zubair and Godbole have reported that unnecessary exploration or negative exploration can be avoided in 20% and 42% cases, respectively [10, 13].

## 6. Conclusion

Our study showed that patients with intra-abdominal testis and vanishing testes benefited from laparoscopy due to the fact that this technique provided them with a definitive diagnosis, direct surgical approach according to the location of testes, and avoidance of unnecessary abdominal exploration in case of vanishing testis. Laparoscopy clearly demonstrates the anatomy and provides visual information upon which a definitive decision can be made.

## Figures and Tables

**Table 1:** 

S. No.	Position of testis on diagnostic laparoscopy	Number
1.	<2 cm from deep ring (low intra-abdominal)	17 (51.5%)
2.	>2 cm from deep ring (high intra-abdominal)	12 (36.3%)
3.	Blind ending vas and vessels	1 (3.03%)
4.	Vas and vessels entering deep ring	3 (9.09%)

**Table 2:** 

Diagnostic laparoscopy findings	Number of testis	Morphology of testis (*n*)	%
High intra-abdominal testis	17	Normal (4)	23.5
		Hypoplastic (10)	58.8
		Atropic (3)	17.6

Low intra-abdominal testis	12	Normal (11)	91.6
		Hypoplastic(1)	9.4

Blind ending vas and vessels	1	No testis found (1)	100

Vas and vessels entering the deep ring	3	Normal testis (3)	100

## Data Availability

The data used to support the findings of this study are included within the article.
